# Improving the quality of COVID-19 care in Sierra Leone: A modified Delphi process and serial nationwide assessments of quality of COVID-19 care in Sierra Leone

**DOI:** 10.1371/journal.pgph.0002670

**Published:** 2023-12-06

**Authors:** Daniel Youkee, Michael Lahai, Abdul R. Mansaray, Sorie Samura, James Bunn, Sulaiman Lakoh, Stephen Sevalie

**Affiliations:** 1 National COVID-19 Emergency Response Centre, Freetown, Sierra Leone; 2 King’s College London, King’s Global Health Partnerships, School of Life Course and Population Health Sciences, London, United Kingdom; 3 Ministry of Health and Sanitation, Freetown, Sierra Leone; 4 College of Medicine and Allied Health Sciences, University of Sierra Leone, Freetown, Sierra Leone; 5 Foreign Commonwealth and Development Office, British High Commission, Freetown, Sierra Leone; 6 34th Military Hospital, Wilberforce, Freetown, Sierra Leone; Amity University Amity institute of public health, INDIA

## Abstract

**Introduction:**

Improving the quality of care that patients receive is paramount to improving patient outcomes and engendering trust during infectious disease outbreaks. Whilst Quality Improvement (QI) is well established to drive improvement in routine care and in health systems, there are fewer reports of its use during infectious disease outbreaks.

**Methods:**

A modified Delphi process was undertaken to create a standardized assessment tool for the quality of COVID-19 care in Sierra Leone. Four rounds of assessment were undertaken between July 2020 and July 2021. To assess change across the four assessment periods compared to baseline we used a mixed effects model and report coefficients and p values.

**Results:**

During the Delphi process, 12/14 participants selected the domains to be assessed within the tool. The final 50 questions included 13 outcome questions, 17 process questions and 20 input questions. A total of 94 assessments were undertaken over four assessment periods at 27 facilities. An increase of 8.75 (p = <0.01) in total score was seen in round 2, 10.67 (p = <0.01) in round 3 and 2.17 (p = 0.43) in round 4 compared to baseline. Mean cumulative scores for COVID-19 Treatment Centres were higher than Hospital Isolation Units (p<0.02) at all four timepoints. Significant improvements were reported in coordination, diagnostics, staffing, infection prevention and control (IPC), nutrition, and vulnerable populations domains, but not in the oxygen, care processes, infrastructure and drugs domains.

**Conclusion:**

We demonstrate the feasibility of creating a quality of care assessment tool and conducting sequential nationwide assessments during an infectious disease outbreak. We report significant improvements in quality-of-care scores in round 2 and round 3 compared to baseline, however, these improvements were not sustained. We recommend the use of QI and the creation of standardised assessment tools to improve quality of care during outbreak responses.

## Introduction

Improving the quality of care that patients receive during outbreaks of infectious disease is paramount to both improving patient outcomes and engendering trust and healthcare seeking. Quality improvement (QI) can be defined as “the use of deliberate and defined methods in continuous efforts to achieve measurable improvements in the efficiency, effectiveness, performance, accountability, outcomes, and other indicators of quality in services or processes” [1]. Whilst QI is well established to drive improvement in health systems and routine care, there are fewer reports of its use during public health emergencies [2] and there “is no reason why the mindset, concepts and tools of improvement and safety science cannot be applied to acute or emergency situations” [3]. During the 2014–2016 West Africa Ebola Epidemic, at times, quality of patient care was neglected [4] and the facilities where patients were cared for were inadequate [5], likely leading to excess mortality and extension of the epidemic. In this article we describe the use of QI and the creation of a standardized assessment tool to assess and improve patient care and patient care facilities during the COVID-19 pandemic in Sierra Leone.

Most reports of the use of QI during the COVID-19 pandemic, are in it’s more traditional sphere, to maintain and improve essential health services [6] and reported from high-income countries [7]. A recent scoping review of QI during COVID-19 found 26 articles, with none conducted in Africa, seven articles focused on QI methods to assess the COVID-19 public health response [7]. Curtis et al applied the Centres for Disease Control US guidelines to assess COVID-19 surveillance for hospitalised patients in Victoria, Australia [8]. A qualitative interview study evaluated the response to Zika virus in Florida, mapped results to the WHO Health Systems Framework and extrapolated the learning to COVID-19 response [9]. Another study assessed preparedness across several countries using the WHO Health System Framework [10]. Another study used annual reporting data from the International Health Regulations to assess preparedness across 182 countries [11]. Whilst Boyce et al describe the creation of a Rapid Urban Health Security Assessment Tool, to assess preparedness for COVID-19 response at the local government level [12]. None of the articles retrieved focused on quality of care at the facility or patient level. Indeed, some authors argue that quality of care was initially overlooked during COVID-19 and that quality of care should be integrated into emergency preparedness and response plans [13].

In Sierra Leone, informed by the previous public health response to Ebola [14], a National COVID-19 Emergency Response Centre (NaCOVERC) including a Case Management (CM) pillar was established at the outset of the Pandemic. The CM Pillar was primarily responsible for the clinical management of suspected and confirmed cases of COVID-19 and published the first Case Management Strategy and Standard Operating Procedures in April 2020 [15]. These guidelines were updated periodically, as new evidence emerged [16]. A QI working group was established within the case management pillar in May 2020, tasked to improve the quality of care for patients with suspected or confirmed COVID-19. In this article, we describe the use of QI to improve quality of care for COVID-19 in Sierra Leone. We describe the creation of a standardised assessment tool for the quality of COVID-19 care and report serial nationwide assessments of quality.

## Methods

### The context: COVID-19 in Sierra Leone

**Fig 1:**
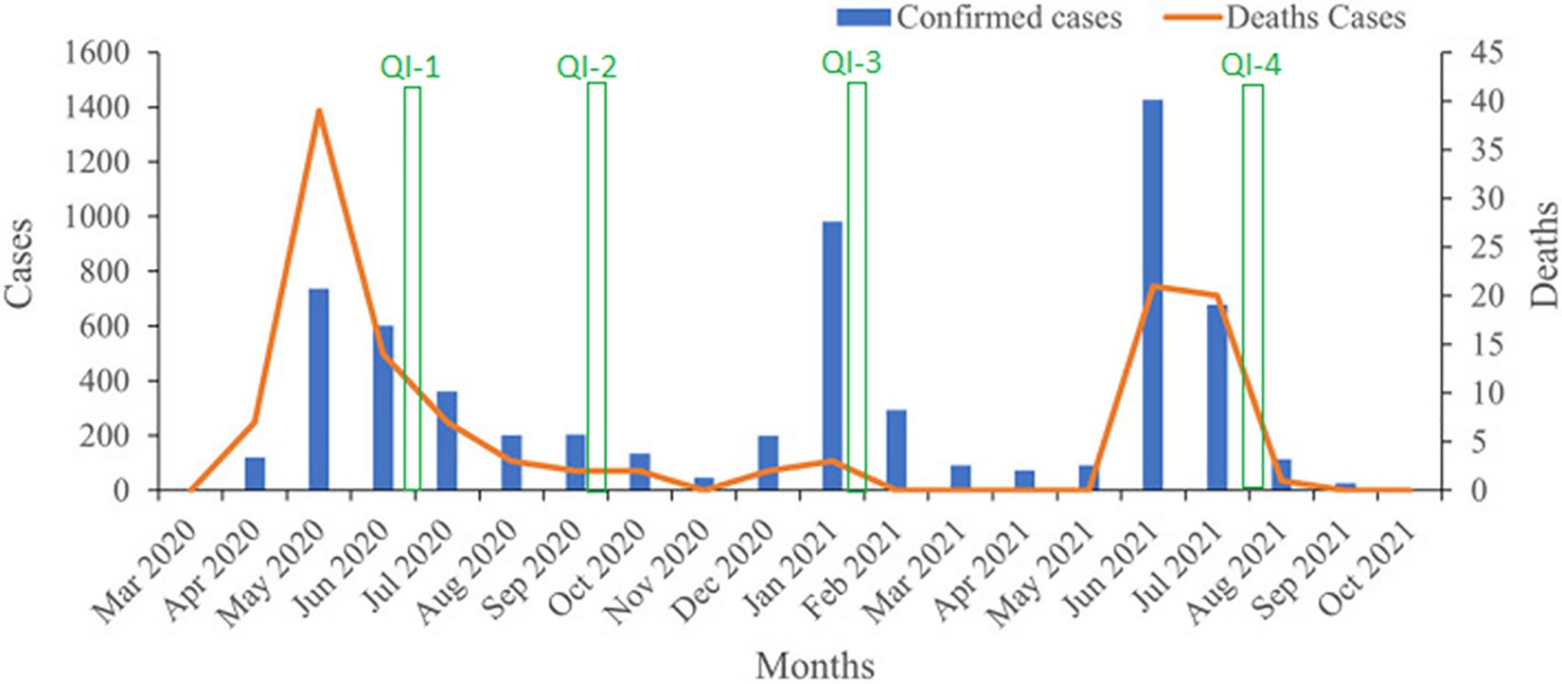
Timeline of COVID-19 case and deaths in Sierra Leone, with the four quality assessment periods indicated in green, adapted from Liu et al [18].

The first COVID-19 case in Sierra Leone was recorded on the 30 March 2020; cases increased to a peak in June 2020 before steadily declining [17]. As of October 31, 2021, a total of 6,398 cases and 121 related deaths had been confirmed in Sierra Leone and a total of 249,534 COVID-19 reverse transcription polymerase chain reaction (RT-PCR) tests had been conducted and the average positivity rate was 2.56%. Three waves of COVID-19 were recorded, occurring during weeks 15–46 in 2020 (2,369 cases), week 47 in 2020 to week 16 in 2021 (1,665 cases), and weeks 17–43 in 2021 (2,364 cases), respectively (Fig 1) [18].

The care of patients with suspected or confirmed COVID-19 was the responsibility of the CM pillar. To provide effective care and reduce the transmission of confirmed or suspected COVID-19 cases, the CM pillar used existing infrastructure to establish COVID-19 treatment centres (CTCs), COVID-19 community care centres (CCCs) and hospital isolation units (HIUs) to manage severe or critically ill patients, asymptomatic or mild COVID-19 cases and suspected COVID-19 cases, respectively. The case management response has previously been described in more detail [17]. A schema demonstrating patient flow between the facilities is provided in *S1 Fig.*

This manuscript describes the use of QI to assess and improve patient care in Sierra Leone in three phases. The first phase was the development of a quality assessment tool, the second phase was the assessment of quality at COVID-19 care facilities and the third phase was statistical analysis of the quality assessment data.

### Phase one: Development of the quality assessment tool

A modified Delphi process was undertaken by the QI technical working group with the objective of creating a standardized assessment of the quality of care at CTCs, CCCs and HIUs. The group was comprised of 14 members with different expertise drawn from governmental and non-governmental agencies. Criteria for participant selection was; expertise in COVID-19 patient care or centre management; experience in outbreak response or experience in quality improvement. The QI working group pre-specified the following criteria for the assessment tool; standardized, in a checklist format; uses mixed methods and triangulates data [19]; possesses good inter-observer reliability; quick to conduct within 2–4 hours; measures “tracer items” [20] or “signal functions” [21]; aimed at the facility level and outputs data in dashboard format that can be easily and correctly interpreted by staff and decision makers. Tool creation was guided by the Donabedian model for evaluating quality of care. The Donabedian model is a conceptual model that provides a framework for evaluating quality of care. The Donabedian framework categorizes information on quality into three categories; outcomes, processes and structures (inputs) [22]. A scoping review of assessment tools and operational guidance [16, 23–26], was conducted to elicit the constructs and domains that comprised quality of care for COVID-19. We did not pre-specify the number of Delphi rounds to be completed, and the process continued until consensus was reached. Consensus was defined as agreement of >80% of the participants. Feedback from the Delphi rounds was quantitative, scoring and ranking of items and qualitative feedback during meetings and via email. Due to the urgency of the task, feedback was not anonymised.

A consultative meeting was held where participants were orientated to the objectives and the pre-specified criteria for the assessment tool, and invited to list the most important domains that constitute quality of care. The first Delphi step followed, where domains were ranked by their importance, and the highest ranked ten domains were maintained. This was an iterative process, where overlapping domains were aggregated. The results were circulated until consensus was reached. In the second Delphi step participants were sent a Microsoft Excel spreadsheet with the ten selected domains including sample items for each domain. Participants were encouraged to submit additional items for each domain and asked to suggest the most feasible and accurate method of ascertaining the answer. This generated an extended list of items which was compiled by the QI working group. The extended list of items was circulated to participants. Participants were asked to rank their top five questions for each domain. Questions were classified as either outcome, process or structure/input questions using the Donabedian framework [22]. The QI working group collated and grouped the methods for answering each item into three categories; direct observation by the assessor [27], retrospective review of patient file or clinical documentation or interview with health facility staff.

### Phase two: Quality assessment

Four nationwide rounds of assessment were undertaken between July 2020 and July 2021. Round 1 from 16/07/20-20/07/20, round 2, from 24/09/20-28/09/20, round 3, from 19/01/2021–25/01/2021, and round four, from 14/07/2021–22/07/2021, see *Fig 1.* Assessment was undertaken by a team of clinical and public health professionals, who were all trained on the use of the tool and administered the tool under supervision until proficient. The assessors were independent observers and were not directly involved or responsible for the quality of care at the facilities assessed. The majority of assessors had participated in the Delphi process to create the quality assessment tool. COVID-19 health facilities and district medical teams were alerted in advance of the visit and permission sought. Four assessment teams covered the following regions; Western Urban and Rural; Southern, Eastern and Northern provinces. Data were collected by direct observation, including entry into the patient area, retrospective reviews of patient file or clinical documentation, and interviews with health facility staff. Data were collected on printed assessment tools and then entered in a Microsoft Excel database.

To rapidly assess inter-observer variability the tool was piloted at one CTC and one HIU on consecutive days by different assessors. Assessment results were compiled centrally by the QI working group and the results were presented as a dashboard. Immediately on completing assessment a verbal report of the results was shared with the facility in charge, and a list of priority actions for facility improvement generated. Compiled assessment results were shared via online meetings with the facility assessed, district case management coordinators, the regional case management leads, the Emergency Operations Centre pillar leads, and relevant donors and implementing partners. National results were compiled and presented in person and online to the Ministry of Health, NaCOVERC and partners using dashboards as shown in S2 Fig.

### Phase three: Statistical analysis

Data were uploaded to STATA v17 for analysis. We excluded facilities that had only one assessment conducted as we could not calculate improvement over time, this included all CCCs. Summary scores were presented by domain and mean total cumulative scores. To assess change in domain scores over time, and difference in scores between CTCs and HIUs, we report the mean difference in scores and paired t-tests. To account for our longitudinal dataset clustered at hospital level, we created multilevel mixed effects linear regression models, controlling for within subject (hospital) dependence. Mixed effect models assessing change from baseline are reported as constant (baseline), and coefficients (change from baseline) and p values (significance) at assessment round 2, 3 and 4. A sensitivity analysis including only facilities included in the first round of assessment was conducted. Missing data was assessed per item and median value replacement was used, due to low levels of missing item data. We utilised median as opposed to mean value replacement as the response scoring options could only be whole numbers.”

### Ethical consideration

This was operational research carried out by the NaCOVERC Case Management Pillar during the COVID-19 Pandemic. No identifiable patient information or data were collected or reported during this research.

## Results

The first Delphi round to decide the domains to be assessed by the assessment tool was responded to by 12/14 participants. The final domains included were; coordination; infrastructure; staffing; infection prevention and control; patient care processes; diagnostics and COVID-19 testing; drugs and consumables; oxygen; nutrition; vulnerable populations and safeguarding. 11/14 participants responded to the second round to prioritise questions within the ten domains. The final 50 questions included 13 outcome questions, 17 process questions and 20 structure or input questions^22^. The final tool included ten domains, with five questions for each domain, each question was scored from 0–2, providing a maximum score of 100. The Delphi process was initiated on the 22/06/20 and the tool was finalised on the 06/07/20 and is displayed here *S1 Tool.*

The pilot cumulative scores of the tool, to assess inter-rater variability were 74 vs 72 for CTCs, 66 vs 63 for HIUs. Mean time to complete the tool was 2 hours and 51 minutes (range: 02:10–03:35).

### Quality results

A total of 94 assessments were included in the analysis over four assessment periods; round 1(n = 19) 16/07/20-20/07/20; round 2 (n = 25) 24/09/20-28/09/20; round 3 (n = 25) 19/01/2021–25/01/2021 and round 4 (n = 25) 14/07/2021–22/07/2021. Assessments were conducted at 27 facilities, 10 CTCs and 17 HIUs. Missing item data was low, 0.49% (23/4700) and median value replacement was conducted.

Mean scores per domain for each round of assessment for all 27 facilities are shown in *Table 1.* The difference in mean total scores increased significantly by 9.3 from round one to round two (P<0.01), increased non-significantly by 1.4 from round two to round three (p<0.64), and then significantly reduced by 7.9 from round three to round four (p<0.03). The highest mean scores were seen in the domains of infrastructure 8.3 (SD:2.1) and IPC 8.3 (SD:1.9) and the drugs domain had the lowest mean score of 6.2 (SD:2.9).

**Table 1:** Summary mean scores by domain and by assessment round for all facilities, paired t-tests compare round of assessment to the previous round.

QI round	Coordination	Diagnostics	Drugs	Staffing	Infrastructure	IPC	Nutrition	Oxygen	Care processes	Vulnerable population	Mean Total	P value
Round 1 N = 19	7.8 (1.3)	5.3 (1.9)	5.7 (2.2)	6.8 (1.2)	8.5 (1.8)	7.2 (2.5)	5.6 (2.2)	6.6 (2.6)	7.8 (2.1)	6.1 (1.6)	67.5 (12.3)	
Round 2 N = 25	8.8 (1.5)	7.0 (2.5)	6.0 (2.5)	7.4 (1.0)	8.7 (1.7)	8.7 (1.5)	8.1 (1.6)	7.5 (2.3)	7.3 (2.0)	7.3 (1.9)	76.8 (10.9)	**0.01**
Round 3 N = 25	8.4 (1.5)	6.9 (1.4)	6.6 (2.5)	7.6 (1.0)	8.9 (1.8)	8.4 (2.0)	7.3 (2.5)	7.8 (2.3)	8.9 (1.1)	7.4 (2.0)	78.2 (9.4)	0.64
Round 4 n = 25	7.4 (1.7)	6.5 (2.0)	6.2 (3.9)	7.2 (1.5)	7.1 (2.5)	8.8 (1.4)	7.0 (2.9)	6.4 (2.3)	8.2 (2.1)	5.6 (2.8)	70.3 (14.3)	**0.03**
Overall mean	8.1 (1.6)	6.5 (2.1)	6.2 (2.9)	7.3 (1.2)	8.3 (2.1)	8.3 (1.9)	7.1 (2.5)	7.1 (2.4)	8.1 (1.9)	6.6 (2.3)	73.6 (12.5)	

A mixed effects model of quality scores compared to baseline is reported in *Table 2*. An increase of 8.75 (p = <0.01) from baseline was seen in round 2, 10.67 (p = <0.01) in round 3 compared to baseline and 2.17 (p = 0.43) in round 4 compared to baseline. In round two significant improvements were seen in coordination, diagnostics, IPC, nutrition and vulnerable population domains. In round three, significant improvements compared to baseline were seen in diagnostics, staffing, IPC, nutrition and vulnerable populations. At round four compared to baseline, significant improvements were seen in diagnostics, IPC and nutrition whilst a significant decrease was seen in infrastructure. Disaggregated results, showing CTCs and HIUs separately are presented in the *S1 and S2 Tables.* Mean cumulative scores for CTCs were higher +5.8 than HIUs (p<0.02) overall and at all four timepoints*, S1 and S2 Tables.* Sensitivity analysis excluding facilities which were not included in round 1 (baseline), reported similar results, *S3 Table*.

**Table 2:** Mixed effect model compares quality of care score at assessment round 2, 3 and 4 to baseline quality of care assessment score in all facilities.

QI round	Coordination Coefficient (p value)	Diagnostics	Drugs	Staffing	Infrastructure	IPC	Nutrition	Oxygen	Care processes	Vulnerable population	Mean Total
Round 1	7.8	5.25	5.68	6.84	8.50	7.30	5.70	6.75	7.80	6.05	67.77
Constant											
N = 19											
Round 2	**0.92 (0.04)**	**1.73 (<0.01)**	0.27 (0.34)	0.60 (0.09)	0.20 (0.72)	**1.43 (<0.01)**	**2.37 (<0.01)**	0.69 (0.23)	-0.47 (0.40)	**1.23 (0.03)**	**8.75 (<0.01)**
coefficient (p value)											
n = 25											
Round 3	0.52 (0.25)	**1.68 (<0.01)**	1.01 (0.19)	**0.76 (0.03)**	0.40 (0.47)	**1.12 (0.02)**	**1.58 (<0.01)**	1.14 (0.05)	1.09 (0.05)	**1.41 (0.01)**	**10.67 (<0.01)**
coefficient (p value)											
n = 25											
Round 4	-0.49 (0.29)	**1.26 (0.02)**	0.51 (0.51)	0.32 (0.38)	**-1.40 (0.01)**	**1.45 (<0.01)**	**1.25 (0.03)**	-0.37 (0.53)	0.37 (0.51)	-0.48 (0.40)	2.17 (0.43)
coefficient (p value)											
n = 25											

## Discussion

Our study describes the creation of a standardised assessment tool for the quality of COVID-19 care and reports serial nationwide assessments of quality. We demonstrate that assessing quality of care during health emergencies is feasible and can be completed in a timely manner. From initiating the creation of the tool to reporting nationwide results took less than a month. There is likely a balance between the time taken to create a tool and the time pressure to establish a baseline and plan improvement of care. In future, pre-prepared tools alongside wider dissemination of tool development methods could further reduce the time taken to establish a baseline of quality of care during infectious disease outbreaks. The low levels of missing item data, suggest the mixed methods used to ascertain data are feasible to undertake at the facility level. The development of assessment tools on electronic tablets and app-based software, may further reduce missing data, reduce transcription errors and reduce data inputting time. Our assessment process achieved good nationwide coverage with all CTCs and HIUs in Sierra Leone being assessed at least twice.

The literature and current tools for COVID_19 largely focus on preparedness rather than quality of care [10, 25, 28, 29]. The Pan American Health Organization Hospital Readiness Checklist for COVID-19 [25] was adapted in Sierra Leone, and the authors report a cross-sectional survey of 9 hospitals. That assessment noted significant gaps in *“COVID-19 leadership*, *coordination*, *health information*, *rapid identification*, *diagnosis*, *isolation and clinical procedures*” [29]. Ogoina et al reports using a modified WHO preparedness tool [25], with 13 domains and 124 indicators, in Nigeria. Twenty of the 68 CTCs in Nigeria responded to the one-off self-assessment [30], with 3 (15%) hospitals reporting adequate readiness. A similar self-assessment in Gondar, Ethiopia using the WHO checklist found only 1 out of 8 assessed hospitals had adequate readiness [31]. In Malawi an existing tool, the Malawi Emergency and Critical Care Survey, developed using Nominal Group Technique process [32] was adapted to rapidly assess baseline readiness at 13 hospitals. The study interviewed 101 clinical staff, triangulating responses from multiple respondents to reduce reporting bias, and found gaps in oxygen access, personal protective equipment and isolation rooms [33]. In our study, we took the next logical step, to move from assessing preparedness to sequentially assessing quality of care. Quality improved significantly from round 1 through to round 3, demonstrating that QI methods can drive improvements in quality of care during an infectious disease outbreak. Our QI intervention was guided by principles for measuring quality of care from the High Quality Health Systems Commission [34]: Accountability and action were key, the Quality working group engaged key accountable people; including the facility in charge; the district medical officer; the regional COVID-19 case management leads and the central case management pillar. Assessment results were disseminated to key accountable individuals and a list of priority actions from each assessment was disaggregated into facility level actions, district level actions, and national level actions, with tasks assigned accordingly. The assessment process was designed to provide timely information to decision makers, with assessment results compiled and presented at the central level and district level within a week of assessment. Results were presented in dashboards, displaying colour coding and cumulative scores, allowing gaps to be identified and good practice to be recognised. Previous outbreak experience [35] and early experience in the pandemic revealed that achieving high quality of care would require a broad set of inputs and processes. This led to the adoption of a broad, holistic view of quality, encompassing staff, space, systems and supplies [36] required to achieve high quality care. This is particularly relevant to the health system in Sierra Leone where it was recognised that a narrow clinical focus would be insufficient to achieve high quality care in a fragmented health system [37] with low numbers of health care workers [38].

The reduction in quality observed from round three to round four may reflect a de-prioritisation of resources to COVID-19 CTCs by the health system [39] due to the reduction in inpatient case load and reported COVID-19 related deaths during that period [18]. This may reflect a healthy re-prioritisation of limited health system resources back towards essential health services [40], however, in the final assessment care was not significantly better than at baseline, and the reduction in the infrastructure domain neither bodes well for future preparedness. The reduction in the infrastructure domain was likely multifactorial; an absence of financing for the maintenance of facilities; a repurposing of facilities back to their original essential health service function as patient admissions declined and a shift of financing towards COVID-19 vaccination efforts instead of patient care.

Mean quality scores were significantly higher for CTCs than HIUs at all timepoints. In the authors collective experience, outbreak response resources and attention are largely concentrated on confirmed cases and treatment centres, to the detriment of suspected cases and HIUs [35], and as a result HIUs are comparatively underinvested in compared to CTCs. HIUs receive patients with suspected disease, with a broader range of underlying disease conditions, requiring a broader range of interventions, equipment and medications. In the capital Freetown, due to periods of significant delay in receiving test results, more COVID-19 deaths occurred in HIUs than in CTCs [41]. Whilst HIUs had lower mean scores at all timepoints, the decrease in mean cumulative scores in the final fourth round of assessment was not statistically significant for HIUs, suggesting some longer term strengthening of HIUs (mainly based in hospitals) may have been achieved in Sierra Leone. This is important as HIUs tend to be the permanent infrastructure of the clinical response compared to CTCs which may be established and then decommissioned as the outbreak wanes. A critical decision in Sierra Leone, in contrast to the Ebola response model, was that NGOs were not allowed to set up stand-alone treatment centres but were asked to support the government to establish HIUs and CTCs within existing government facilities.

The domains of drugs and care processes were resistant to improvement and analysis of individual items highlights some areas where improvement did not occur. Whilst NaCOVERC did obtain and distribute medications for COVID-19, these did not routinely include drugs for common co-morbidities, measured by the assessment tool, such as hypertension and diabetes. Drug distribution may not always have reached the facility level, and the lack of access to medications for common co-morbidities remains concordant with reports from facilities [41]. Patient care processes saw little improvement, and indicators with persistent low scores were risk stratification and acuity-based triage of patients. Oxygen scores only showed a modest improvement, this reflects the lack of regular access to high flow oxygen outside of Freetown and inappropriate oxygen prescription and use. Oxygen access in Sierra Leone was severely limited, throughout the outbreak, whilst oxygen cylinders from a private supplier were distributed by NaCOVERC, these did not meet demand and the scale-up of oxygen producing capacity at government hospitals [42] only began in 2022.

Our study has limitations and strengths. Our first assessment took place in June 2020, two months after the first case of COVID-19 was reported in Sierra Leone. It is not therefore, a pre-COVID baseline and it does not capture the initial interventions in the health system, including initial infrastructure investments. Our process used expert opinion and is therefore limited by the experts who were included in the process. Delphi process participants were from a diverse range of clinical and non-clinical backgrounds; however, we could have included a larger group of experts and patients in the process. Due to the urgency of the task, we did not anonymise participant feedback, and this may have affected participant responses. A further limitation of our study, is that there was insufficient time to comprehensively assess the inter-observer variability of our tool, which was only measured on two occasions, but reflected the need for rapid deployment and use of the tool. Finally, our study was not designed to assess the relationship between quality scores and patient outcomes which are the end goal of the process. Our study benefited from using a clear methodology to design the assessment tool, grounded by a conceptual framework [22]. We used multiple overlapping methods of data collection; direct observation; interview with healthcare workers and review of clinical documentation. We report good coverage of the assessment, assessing all CTCs and HIUs in the country at least twice, and are able to report serial assessments over one year. Further work should include greater piloting of assessment tools before their implementation and the inclusion of patients in the Delphi process if feasible.

Based off our experience, we advocate for the standardized assessment of quality of care and use of QI methods during health emergencies. Assessment tools should be designed using appropriate methodology, provide standardized guidance on assessment methods and how to modify tools. Assessments of preparedness, should be designed with an increased focus on quality of care, to enable serial assessments over time, and be grounded in a conceptual theory of change or QI methodology. Standardized assessment of quality of care; allows standards to be agreed and set; encourages assessment of respectful care of an agreed quality; strengthens the rights of the patient; enhances protection of healthcare workers; enables performance to be compared locally, internationally or over time [43, 44]; identifies critical needs; and targets rapid interventions and investments [45]. Finally, standardized assessment of quality of care of patients during health emergencies could be a vital tool to promote learning health systems [46], encourage the sharing of good practice and thereby strengthen global health security.

## Conclusion

This study demonstrates that a quality of care assessment tool can be rapidly created using a modified Delphi process, and that QI methods can drive improvements in care during a health emergency. We recommend that standardized assessments of quality of care and use of QI methods should be incorporated into future health emergencies.

## Supporting information

S1 Fig Schema demonstrating COVID-19 facility types and COVID-19 patient flow in Sierra Leone. (TIF)

S2 Fig Dashboard of COVID-19 treatment centre quality of care scores in round one, used to disseminate assessment results. (TIF)

S1 Table Mixed effect model compares quality of care score at assessment round 2, 3 and 4 to baseline quality of care assessment score in COVID-19 treatment centres. (DOCX)

S2 Table Mixed effect model compares quality of care score at assessment round 2, 3 and 4 to baseline quality of care assessment score in hospital isolation units. (DOCX)

S3 Table Sensitivity analyses including only facilities that were included in the first assessment round, mixed effect model compares quality of care score at assessment round 2, 3 and 4 to baseline quality of care assessment score. (DOCX)

S1 Tool Finalised quality of COVID-19 care assessment tool. (XLSX)

S1 Data Anonymised data file. (CSV)
