# Supplementary figures and images for: Improving the quality of COVID-19 care in Sierra Leone: A modified Delphi process and serial nationwide assessments of quality of COVID-19 care in Sierra Leone
Source: PLOS Glob Public Health. 2023 Dec 6;3(12):e0002670. doi: 10.1371/journal.pgph.0002670 (PMC10699596; doi:10.1371/journal.pgph.0002670)

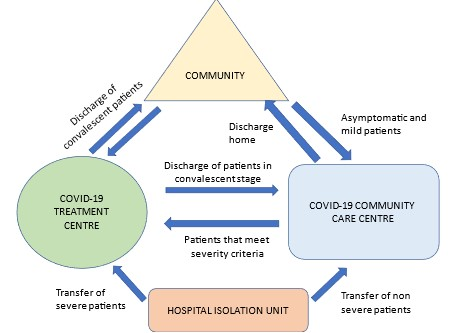

Supplement: S1 Fig — (TIF) [file pgph.0002670.s001.tif]

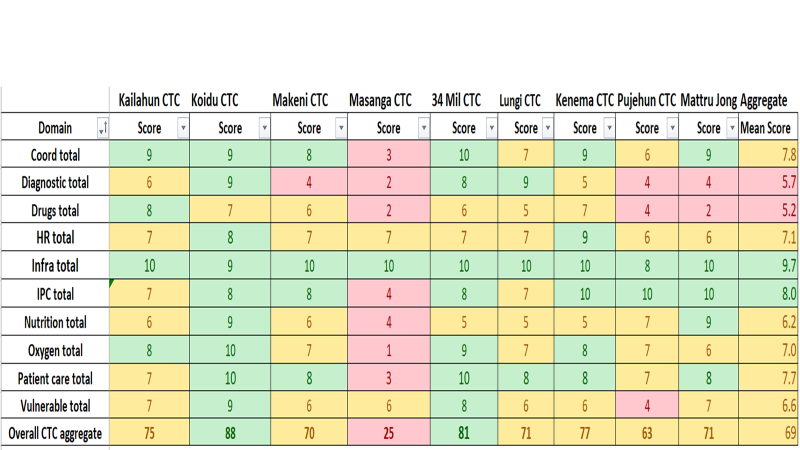

Supplement: S2 Fig — (TIF) [file pgph.0002670.s002.tif]
