# Supplementary material for: Improving the quality of COVID-19 care in Sierra Leone: A modified Delphi process and serial nationwide assessments of quality of COVID-19 care in Sierra Leone
Source: PLOS Glob Public Health. 2023 Dec 6;3(12):e0002670. doi: 10.1371/journal.pgph.0002670 (PMC10699596; doi:10.1371/journal.pgph.0002670)
Supplement: S1 Table — (DOCX) [file pgph.0002670.s003.docx]

| QI round | Coordination | Diagnostics | Drugs | Staffing | Infrastructure | IPC | Nutrition | Oxygen | Care processes | Vulnerable population | Mean Total |
| --- | --- | --- | --- | --- | --- | --- | --- | --- | --- | --- | --- |
| Round 1  constant | 8.29 | 6.38 | 6.13 | 7.29 | 9.57 | 8.34 | 6.49 | 8.03 | 8.29 | 6.57 | 75.06 |
| Round 2  Coefficient (p value) | 1.11 (0.12) | 0.42 (0.58) | 0.57 (0.59) | 0.31 (0.60) | -0.37 (0.65) | 0.56 (0.32) | 1.41 (0.08) | 0.37 (0.61) | 0.82 (0.23) | 1.23 (0.13) | 7.74 (0.11) |
| Round 3  Coefficient (p value) | 0.38 (0.61) | 0.60 (0.44) | 0.29 (0.79) | 0.16 (0.80) | -0.79 (0.34) | 0.88 (0.13) | **1.93 (0.02)** | 1.03 (0.16) | 1.27 (0.07) | **2.09 (0.01)** | 8.32 (0.06) |
| Round 4  Coefficient (p value) | -0.99 (0.17) | 0.12 (0.88) | 1.97 (0.06) | 0.11 (0.85) | **-2.17 (<0.01)** | 0.56 (0.32) | 1.11 (0.16) | -1.43 (0.05) | -0.49 (0.47) | -0.37 (0.65) | -1.26 (0.77) |

*S1 Table: Mixed effect model compares quality of care score at assessment round 2, 3 and 4 to baseline quality of care assessment score in* ***COVID-19 Treatment Centres***
