# Supplementary material for: Improving the quality of COVID-19 care in Sierra Leone: A modified Delphi process and serial nationwide assessments of quality of COVID-19 care in Sierra Leone
Source: PLOS Glob Public Health. 2023 Dec 6;3(12):e0002670. doi: 10.1371/journal.pgph.0002670 (PMC10699596; doi:10.1371/journal.pgph.0002670)
Supplement: S2 Table — (DOCX) [file pgph.0002670.s004.docx]

| QI round | Coordination | Diagnostics | Drugs | Staffing | Infrastructure | IPC | Nutrition | Oxygen | Care processes | Vulnerable population | Total score |
| --- | --- | --- | --- | --- | --- | --- | --- | --- | --- | --- | --- |
| Round 1  Constant | 7.53 | 4.60 | 5.43 | 6.59 | 7.89 | 6.64 | 5.19 | 5.97 | 7.50 | 5.67 | 63.03 |
| Round 2  Coefficient (p value) | 0.80 (0.14) | **2.52 (<0.01)** | 0.03 (0.97) | 0.74 (0.08) | 0.49 (0.51) | **1.96 (<0.01)** | **3.02 (<0.01)** | 0.87 (0.29) | -1.37 (0.05) | 1.29 (0.09) | **10.24 (<0.01)** |
| Round 3  Coefficient (p value) | 0.66 (0.21) | **2.26 (<0.01)** | 1.44 (0.14) | **1.10 (0.01)** | 1.09 (0.13) | 1.28 (0.06) | 1.44 (0.06) | 1.26 (0.12) | 1.0 (0.14) | 1.15 (0.13) | **12.94 (<0.01)** |
| Round 4  Coefficient (p value) | -0.14 (0.80) | **1.93 (<0.01)** | -0.49 (0.62) | 0.41 (0.34) | -0.99 (0.18) | **1.99 (<0.01)** | 1.36 (0.08) | 0.32 (0.69) | 0.9 (0.19) | -0.50 (0.51) | 4.64 (0.19) |

*S2 Table: Mixed effect model compares quality of care score at assessment round 2, 3 and 4 to baseline quality of care assessment score in* ***Hospital Isolation Units***
