# Supplementary material for: Improving the quality of COVID-19 care in Sierra Leone: A modified Delphi process and serial nationwide assessments of quality of COVID-19 care in Sierra Leone
Source: PLOS Glob Public Health. 2023 Dec 6;3(12):e0002670. doi: 10.1371/journal.pgph.0002670 (PMC10699596; doi:10.1371/journal.pgph.0002670)
Supplement: S3 Table — (DOCX) [file pgph.0002670.s005.docx]

| QI round | Coordination  Coefficient (p value) | Diagnostics | Drugs | Staffing | Infrastructure | IPC | Nutrition | Oxygen | Care processes | Vulnerable population | Total Score |
| --- | --- | --- | --- | --- | --- | --- | --- | --- | --- | --- | --- |
| Round 1  Constant  N=19 | 7.8 | 5.3 | 5.7 | 6.8 | 8.5 | 7.2 | 5.6 | 6.6 | 7.8 | 6.1 | 67.5 |
| Round 2  coefficient (p value)  n=19 | **1.4 (p=0.001)** | **2.1 (p<0.001)** | 0.7 (p=0.42) | 0.6 (p=0.08) | 0.2 (p=0.78) | **1.4 (p=0.01)** | **2.5 (p<0.001)** | 0.50 (p=0.43) | -0.1 (p=0.81) | 1.0 (p=0.08) | **10.0**  **(P<0.001)** |
| Round 3  coefficient (p value)  n=19 | 0.3 (p=0.44) | **1.8 (p<0.01)** | 0.7 (p=0.38) | 0.4 (p=0.21) | 0.4 (p=0.48) | 0.7 (p=0.22) | **1.4 (p=0.03)** | 0.6 (p=0.32) | 0.98 (p=0.12) | **1.6 (p<0.01**) | **9.0**  **(p<0.001)** |
| Round 4  coefficient (p value)  n=19 | -0.7 (p=0.11) | **1.3 (p=0.03)** | 0.6 (p=0.46) | 0.3 (p=0.38) | **-1.5**  **(p<0.01)** | **1.6 (p<0.01)** | 1.3 (p=0.05) | -0.1 (p=0.94) | -0.1 (p=0.96) | -0.5 (p=0.43) | 2.1 (p=0.42) |

*S3 Table: Sensitivity analyses including only facilities that were included in the first assessment round, mixed effect model compares quality of care score at assessment round 2, 3 and 4 to baseline quality of care assessment score.*
